# Retinal vein and artery occlusion as the first manifestation of primary antiphospholipid syndrome in a pediatric patient

**DOI:** 10.5935/0004-2749.2021-0431

**Published:** 2022-07-04

**Authors:** Marina Delgado João, Jorge Vasco Costa, Gil Calvão Santos, Ricardo Dourado Leite, Sandra Guimarães

**Affiliations:** 1 Ophthalmology Department, Hospital de Braga, Braga, Portugal; 2 Ophthalmology Department, Hospital-Escola da Universidade Fernando Pessoa, Gondomar, Portugal

**Keywords:** Antiphospholipid syndrome/complications, Retinal vein occlusion, Retinal artery occlusion, Mycoplasma infection, Humans, Case report, Síndrome antifosfolipídica/complicações, Oclusão da veia retiniana, Oclusão da artéria retiniana, Infecção por *Mycoplasma*, Humanos, relato de caso

## Abstract

Antiphospholipid syndrome is an acquired autoimmune disease characterized by hypercoagulability associated with recurrent venous and arterial thromboembolism in the presence of antiphospholipid antibodies. Herein, we report a case of rapid sequential retinal vein and artery occlusion as the first manifestation of a primary antiphospholipid syndrome triggered by an acute *Mycoplasma* infection in a previously healthy 11-year-old patient. On day 1, ophthalmoscopy revealed a central retinal vein occlusion. The patient developed temporal branch retinal artery occlusion the next day. On day 3, a central retinal artery occlusion was observed. Serum lupus anticoagulant, immunoglobulin (Ig) G anticardiolipin, IgG anti-β2-glycoprotein 1 antibody, and *Mycoplasma pneumoniae* IgM antibody levels were increased. Thus, retinal vascular occlusions can be the first manifestation of primary antiphospholipid syndrome. Although it may not improve visual prognosis, prompt diagnosis and treatment are essential to avoid further significant morbidity.

## Abstract (pt)

A síndrome antifosfolipide é uma doença autoimune adquirida caracterizada por hipercoagulabilidade associada a tromboembolismo venoso e arterial recorrente na presença de anticorpos antifosfolipídicos. Aqui, relatamos um caso clínico de oclusão sequencial de veia e artéria da retina como primeira manifestação de uma síndrome antifosfolipíde primária desen­cadeada por uma infeção aguda por *Mycoplasma* num paciente de 11 anos previamente saudável. No primeiro dia, a oftalmoscopia revelou uma oclusão da veia central da retina. No dia seguinte, o paciente desenvolveu uma oclusão do ramo temporal da artéria central da retina. No terceiro dia, uma oclusão da artéria central da retina foi diagnosticada. Os níveis de anticoagulante lúpico sérico, anticorpos IgG anticardiolipina e IgG anti-β2-glicoproteína 1 e anticorpos IgM para *Mycoplasma pneumoniae* estavam aumentados. As oclusões vasculares retinianas podem ser a primeira manifestação da síndrome antifosfolipíde primária. Apesar do prognóstico visual ser reservado, o seu diagnóstico e o tratamento imediatos são essenciais para evitar outras morbilidades associadas.

## INTRODUCTION

Antiphospholipid syndrome (APS) is an acquired autoimmune disease associated with recurrent thromboembolism and persistently elevated levels of antibodies directed against membrane anionic phospholipids (anticardiolipin antibody, antiphosphatidylserine antibody) or their associated plasma proteins, predominantly beta-2 glycoprotein I, or evidence of a circulating anticoagulant^(1)^. APS can develop any time from the neonatal period to adolescence^(1)^. APS can be associated with an underlying systemic autoimmune condition, such as systemic lupus erythematosus, or can occur as an isolated form called primary APS^(2)^. In primary APS, the mean age of disease onset is lower than that in patients with autoimmune disease-associated APS (8.7 years vs. 12.7 years)^(2)^. Both arterial and venous occlusions can occur; however, venous thromboembolism is the most frequent complication (increased risk of up to 10-fold^(3)^) and might occur in any vascular bed. Ocular manifestations include central (CRVO) and branch retinal vein occlusions, central (CRAO) and branch (BRAO) retinal artery occlusions, choroidal occlusions, anterior and posterior ischemic neuropathy, amaurosis fugax, and diplopia^(2,4-6)^.

We report a rare case of sequential CRVO and CRAO in a pediatric patient diagnosed with primary APS triggered by an acute *Mycoplasma* infection.

## CASE REPORT

**Figure 1:**
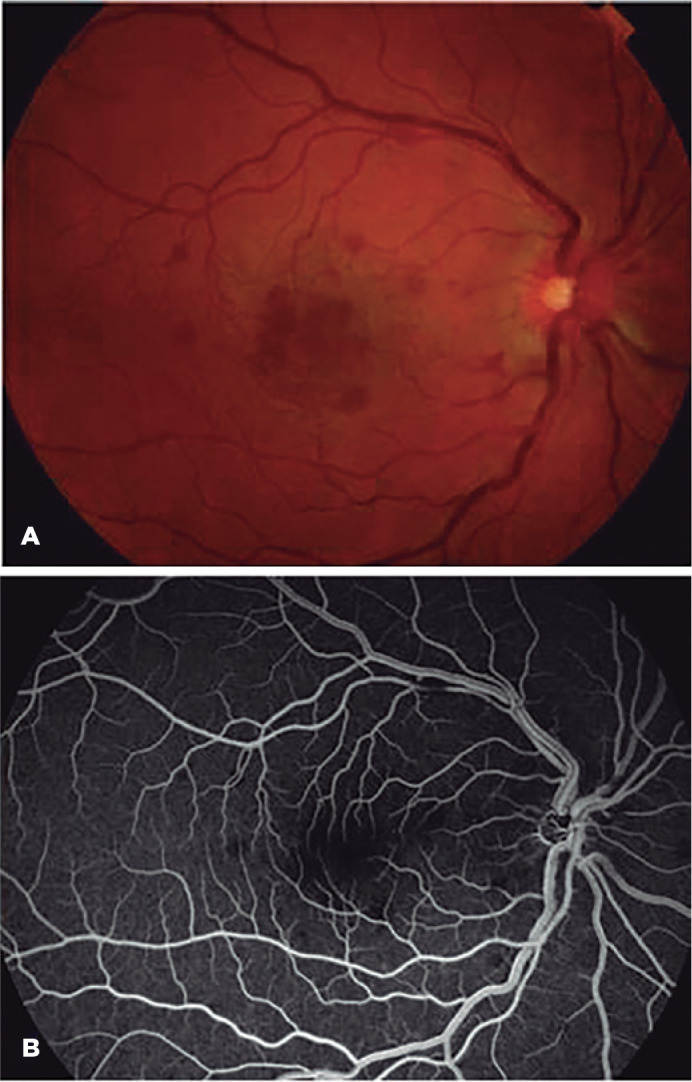
At presentation, color fundus photography (A) and fluorescein angiography (B) of the right eye showed a delayed filling of the venules, venous congestion, and scattered retinal hemorrhages consistent with a nonischemic central retinal vein occlusion.

**Figure 2:**
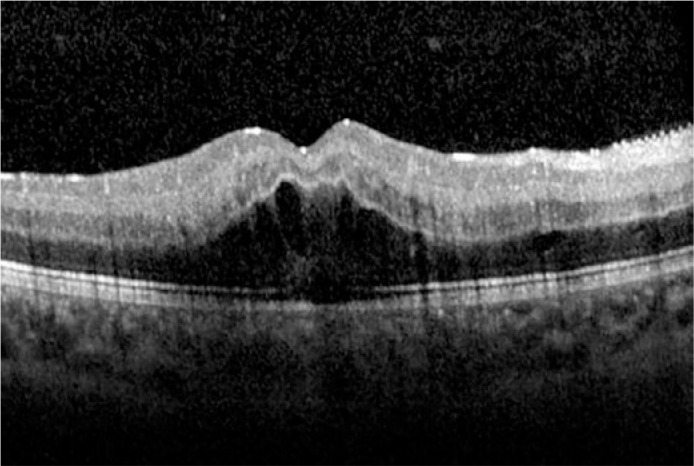
Spectral-domain optical coherence tomography through the fovea of the right eye at presentation showed cystoid retinal edema.

An 11-year-old boy presented to our department with blurry vision in his right eye since he woke up. He denied any ocular pain; however, his parents reported a weight loss over the previous 6 months and persistent cough and mild headache in the previous week. The patient had a history of vernal conjunctivitis and was prescribed topical cyclosporine and corticosteroids. The best-corrected visual acuity (BCVA) was counting fingers (CF), and a relative afferent pupillary defect of the affected eye was observed. Intraocular pressure was 11 mm Hg. Biomicroscopy exam was normal. Right eye fundoscopy revealed optic disc edema, tortuous dilated retinal veins, retinal hemorrhages, and macular edema (ME). Color fundus photography, spectral-domain optical coherence tomography (SD-OCT), and fluorescein angiography (FA) were performed. FA confirmed the diagnosis of CRVO (Figure 1). SD-OCT showed cystoid ME (Figure 2). Therefore, the patient was admitted for further investigation.

**Figure 3:**
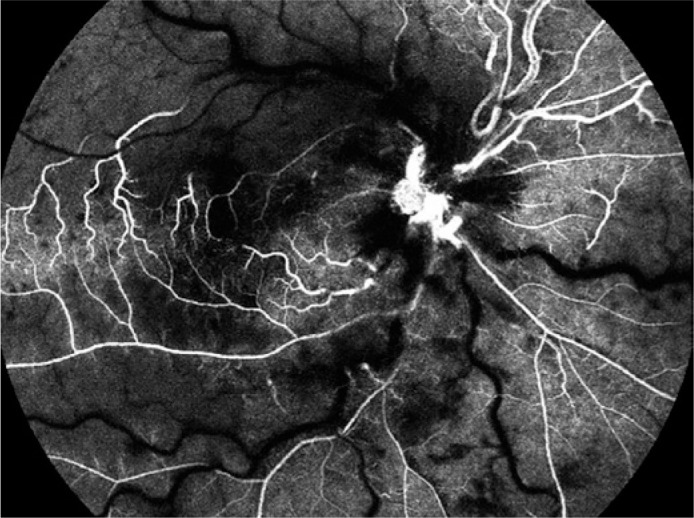
On day 3, fluorescein angiography showed central retinal artery occlusion with cilioretinal artery sparing despite anticoagulation treatment.

On day one, superior temporal BRAO was observed and confirmed with FA. Anticoagulation treatment with low molecular-weight heparin and systemic corticosteroids was initiated; however, the patient presented with subjective vision worsening on day 3. BCVA decreased to light perception in the affected eye. On fundoscopy, a CRAO with cilioretinal artery sparing was suspected and confirmed with FA (Figure 3).

Extensive diagnostic testing was performed, and evaluations were obtained from pediatric, immunochemotherapy, neurology, and rheumatology departments. A complete blood count, metabolic panel, and urinalysis were normal. A coagulation profile revealed a slightly raised activated partial thromboplastin time of 38.8 seconds and normal thrombin and prothrombin times. Antinuclear antibody level, antineutrophil cytoplasmic antibody level, rheumatoid factor, and erythrocyte sedimentation rate were normal. Factor V Leiden, factor II, and methylenetetrahydrofolate reductase (MTHFR) were negative; however, fibrinogen levels were elevated. Serum lupus anticoagulant (ratio mix of 2,62), immunoglobulin (Ig) G anticardiolipin (280 U/ml), and IgG anti-β2-glycoprotein 1 (867 UA) levels were increased.

Blood cultures and serology studies excluded syphilis, tuberculosis, human immunodeficiency virus, herpesvirus (HSV), cytomegalovirus (CMV), Epstein-Barr virus, toxoplasmosis, Bartonella disease, and Lyme’s disease, but revealed *Mycoplasma pneumoniae* IgM antibodies.

Brain and orbits computed tomography and angiography did not show vascular occlusions or other abnormalities. An echocardiogram revealed no cardiac vegetations or foramen ovale, and computed tomography angiography of the carotid vessels did not show abnormalities as well.

Based on clinical and laboratory findings, a presumptive primary APS diagnosis was made, and anticoagulation treatment with warfarin was maintained indefinitely. Tests were repeated 12 weeks later to confirm the diagnosis.

Within the 12 months of the follow-up, there were no signs of ocular complications, such as neovascularization in the retina or the iris, or other systemic thromboembolism. BCVA improved to CF, IOP was 15 mmHg, and SD-OCT confirmed optic disc temporal atrophy and inner retinal layers atrophy in the macula.

## DISCUSSION

Previous reports described pediatric APS patients’ arterial and venous retinal occlusions ^(4-7)^. However, to the best of our knowledge, this is the first case in which an acute *Mycoplasma* infection triggered the sequential retinal vein and artery occlusion as the first manifestation of primary APS. Different infectious agents can induce autoimmunity, and bacterial and viral infections precede some APS cases. Moreover, the association between *Mycoplasma* and APS has been previously reported^(8)^. Molecular mimicry between the infectious agents and the antigenic targets is believed to be the central mechanism by which these agents trigger autoimmunity in genetically predisposed patients^(1)^. Superantigens, such as those produced by *Mycoplasma*, activate T cells expressing particular Vβ gene segments specific for a self-antigen. Therefore, generated autoimmune cross-reactions with host structures can cause tissue damage^(8)^.

APS pathophysiology is incompletely understood, and we do not know enough about the differences in the pediatric population. However, children frequently miss the thrombotic risk factors observed in adults, suggesting a more severe molecular drive capable of breaking the natural antithrombotic mechanisms^(9)^. Retinal vein and artery occlusion are considered rare APS manifestations. A previous study reported that APS patients who developed retinal vascular occlusions have higher risk aPL profiles (triple aPL positivity)^(10)^, as described in our report.

The role of immunosuppressive treatment is uncertain. Despite limited specific data about pediatric APS after a thrombotic event, the current recommendation is to treat a patient with long-term anticoagulation (INR goal of 2-3)^(9)^. Recent observational studies have found that some patients are treated with corticosteroids in addition to other immunosuppressants; however, there is no clear evidence to support their routine use in clinical practice^(9)^.

Visual prognosis in cases of combined CRVO and CRAO is reserved. We adopted a conservative approach with close follow-up. ME eventually resolved, and no neovascularization was noted.

This report highlights the importance to perform a complete and thorough investigation of retinal vessels occlusions in pediatric patients while collaborating with other departments to make a correct diagnosis. Even though it does not improve visual prognosis, prompt treatment is essential to avoid further significant primary APS-associated morbidity.
